# Sustainability, profitability, and resiliency of the fast fashion industries during a pandemic

**DOI:** 10.1177/0958305X241239896

**Published:** 2024-03-20

**Authors:** Meriem Meskini, Tanvir S. Mahmud, Sagar Ray, Amy Richter, Tahlima T. Sithi, Kelvin Tsun Wai Ng

**Affiliations:** 1 Environmental Systems Engineering, 6846 University of Regina, Regina, Canada

**Keywords:** Clothing and textile waste, fast fashion, environmental sustainability, profitability and sales revenue, business characteristics, regression analysis

## Abstract

The size of the global textile market was estimated at nearly one trillion USD in 2021 and the circularity of fashion items are of utmost practical and economic interests. Many postconsumer textile wastes are not recycled, and are sent to landfills for permanent disposal. This study examines the profitability of the selected fashion companies and compares the financial performance through weighted average net profit margin and business characteristics. The 10 companies are divided into 2 groups (conventional and sustainability) for analysis. The conventional fashion companies have a total sales revenue approximately 23 times higher than that of the sustainability fashion companies. The weighted average net profit ratio of the conventional fashion industry averaging +4.7 during the 5-year study period, much higher than the sustainability fashion group. Sustainability fashion industry is emerging and appears more sensitive to fluctuations in business expenses and COVID lockdowns. Net profit was negative for the sustainability group in 2022, and more aids such as government initiatives and incentive programs may be critical to the growth of the sustainability fashion sector. Both sustainability and conventional groups have positive relations between net profit and number of employees and net profit and market shares, respectively. The results suggest that fashion industry has opportunities to be more profitable by adopting socially responsible goals.

## Introduction

Fast fashion refers to the production of low-cost, trendy clothing that moves quickly from design to retail stores to meet consumer demand for the latest fashion trends.^1–3^ However, mass production and rapid growth models promote a consumer mentality and cause public concern about their potential impacts on environment and society.^4–6^ Awareness of environmental sustainability is increasingly popular,^7–9^ and the recent UN Climate Change Conference of 2023 (COP28) has recognized the urgency of our actions on climate change. Fast fashion's unsustainable practices, including its high resource usage, production of textile waste, and exploitation of its workforce, have prompted calls for the sector to adopt more environmentally friendly practices.^10–12^ The global fashion industry is in a constant state of evolution, driven by the rising demand for affordable clothing.^13,14^ The size of the global textile market was estimated at US$ 993.6 billion in 2021 and is expected to expand at a compound annual growth rate of 4.0% from 2022 to 2030.^15^ The circularity of fashion items and the overall sustainability of the fashion industry are thus of utmost practical and economic interests.

### End-of-life clothing waste disposal

Due to their affordable nature, fast fashion items last much shorter and wear out quicker than conventional clothing and accessories, impacting their recyclability.^16,17^ Consequently, the fast fashion industry has produced a considerable amount of postconsumer textile waste, challenging the conventional waste management system.^18,19^ According to McQueen et al.,^20^ only about 15% of textile waste in the United States was recycled in 2017, and the remainder was mostly sent to landfills for permanent disposal. Although landfill design is getting increasingly sophisticated,^21,22^ land disposal continued to contribute greenhouse gas emissions,^23,24^ and groundwater contamination.^25,26^ Many consider landfilling as one of the least desirable options in a waste management hierarchy^27–29^ and the current way to dispose textile waste is not sustainable.

### Sustainability and profitability on fast fashion industry

Fast fashion items are affordable trend-driven garments and are often perceived by consumers as poorer quality.^30^ As such, selling, donation, and repurposing of fast fashion items are often difficult due to the intended short design lifespan of the merchandises, as well as the complex socio-economical consumer behaviors.^31,32^ The fast fashion industry prioritizes cost efficiency in their supply chains to maintain low prices.^33,34^ However, long-term operating savings and green marketing may result from sustainable practices such as employing eco-friendly products and ethical sourcing.^35^ The initial cost of adopting sustainable practices may be higher, but as resource efficiency and waste reduction improve, costs may eventually fall.^36^ Moreover, as awareness of environmental and social issues grows, consumers are increasingly seeking out sustainable and ethical fashion options.^37^ Brands that embrace sustainability can attract a growing segment of eco-conscious consumers, which can expand the customer base, leading to increased sales, and brand loyalty.^35,38^ The often-conflicting effects of environmental, social, and governance (ESG) initiatives on business profits and resiliency were being investigated in various sectors.^39–41^ Fast fashion's traditional model of producing large quantities of cheap, disposable clothing items may face challenges in the long term due to resource constraints and changing consumer preferences.^42^ However, only very few studies are available in this field.

Sustainability appears to have a positive relationship with profitability in the fast fashion industry according to the earlier studies.^1,43^ A study on the Indian fashion market, Bodhanwala and Bodhanwala^44^ claimed that corporate sustainability strategies are beneficial, and these sustainability goals positively correlated to profitability. Jung et al.^45^ also found a positive relationship between sustainability and profitability in the fast fashion industry. Laura et al.^46^ claimed that purchasing decisions can be positively impacted by a product's sustainability, especially when it uses recycled materials to create a piece of clothing. On the contrary, López et al.^47^ reported a negative relationship between sustainability and profitability in 110 firms in Europe using the Dow Jones sustainability index. Huang,^48^ however, found no obvious relationship between sustainability and profitability across 297 electronic companies in Taiwan. Cayaban et al.^49^ studied that sustainability negatively affected the customer intention to buy fast fashion among Filipino consumers. It appears the relationships between sustainability and profitability are complex and more research is needed.

### Objectives and novelty

While there might be initial investments and adjustments required to adopt sustainable practices, many fashion companies are realizing that integrating sustainability into their business models can lead to improved profitability, brand reputation, and long-term viability. The study objectives are to (i) examine the profitability of the selected fashion companies reported by the *Wall Street Journal (WSJ)*, and to (ii) compare the financial performance through weighted average net profit margin and business characteristics between the sustainability fashion industry and the fast fashion industry. It is hypothesized that fast fashion has more opportunities to be more profitable by adopting socially responsible goals. Adopting sustainable strategies as a code of ethics in a business entity helps to establish a reputation and promote brand recognition,^50^ improving long-term profitability. To the best knowledge of the authors, this is the first study to explicitly correlate the average net profit margin and business characteristics of fast fashion companies. Given the rapid growth of the fast fashion industry and the popularity of ESG issues across the globe, this case study provides insights to fashion designers, policy makers, and the waste management professionals. Recent COVID-19 waste studies^51–53^ suggest different waste recycling and disposal behaviors during the lockdowns, this study will shed some lights on the changes of consumer behaviors and spending habits on clothing.

## Methodology

### Selection of the companies

Reliable and verifiable long-term financial records of some of the fast fashion companies are publicly unavailable or difficult to found. WSJ has a long history of providing reliable business and financial data,^54–56^ and is commonly adopted in financial research studies.^57,58^ With a global network of reporters and correspondents, WSJ provides in-house coverage in multiple regions. WSJ also covers a wide range of business and financial topics, including global markets, corporate news, economic developments, and advances in technology. We focused exclusively on fashion industries for which financial data was accessible through WSJ to ensure consistency in data. The selected study period is from 2018 to 2022, covering the COVID-19 period. The global pandemic and the associated lockdowns have affected many aspects of our lives, including how we generate, recycle, and dispose municipal solid waste.^51,59,60^

A total of 10 fashion companies with operations in multiple countries have been carefully selected from the WSJ list. All of the selected companies must submit annual data to WSJ and have publically available ESG reports. Table 1 summarizes the selected companies in this study. The companies are classified into two equal-number groups, the “sustainability fashion industry” group and the “conventional fashion industry” group. The classification is based largely on the published ESG reports and the public perception of the companies. It is important to note that almost all selected companies have published specific reports and/or ESG policies related to environmental sustainability or have publicly supported various sustainability projects.

**Table 1.** The selected fashion companies and their key business area.

	Company	Known business area	Headquarter
Sustainability fashion companies	Thredup	Recommerce	United States
	Carbios	Biochemistry	France
	Sulzer	Industrial engineering and manufacturing	Switzerland
	Renewcell	Recycling	Sweden
	Lenzing	Recycling	Australia
Conventional fashion companies	GAP	Retail	United States
	AEO	Retail	United States
	DIOR	Retail-Luxury goods	France
	H&M	Retail	Sweden
	Esprit	Retail-Fashion	Hong Kong

AEO: American Eagle Outfitters.

The five selected companies from the “sustainability fashion industry” group are Sulzer, Carbios, Renewcell, Lenzing, and Thredup. All of these companies have emphasized green policy and environmental sustainability in their websites and publicly available reports. These companies have explicitly stated their company goals of reducing textile waste and promoting a shift to a more sustainable textile industry, though their main business area may not necessarily be limited to fashion only (Table 1). According to the information provided by the companies, all of them are actively promoting circular economy practices, and developing eco-friendly products and solutions to contribute to a more sustainable future. Compared to the conventional fashion companies, the selected five companies appear to have a more forward-thinking and proactive approach to waste minimization and circular economy development with respect to clothing and textile waste.

The five selected companies from the conventional group are H&M, GAP, Esprit, American Eagle Outfitters (AEO), and Christian Dior (CD). We have specifically selected companies with diverse clienteles, from youth to high-end customers (Table 1). All selected companies are known to be responsive to fashion trends and have well-established international supply chains. For instance, in the 2000s, H&M and ZARA started introducing 52 “micro-seasons” to the market each year, the equivalent of a new collection every week.^61^ Christian Dior, a multinational luxury fashion house, is less active on recyclability project that aims to minimize the waste derived from the brand's products.^6^) According to Serdaroglu,^63^ GAP contributes approximately 50 tonnes of its produced textiles disposed in landfills each year, out of which 39% is not biodegradable. Similar to many other fashion companies not considered, these fashion companies contribute to the generation of clothing and textile wastes.

### Financial data collection and regression analysis

As discussed, the financial information used to quantify industrial profitability is derived from the publicly available WSJ annual financial reports. Since the size of these companies are different, specific weights based on its market share or contribution to total sales in that industry were assigned to each selected company. This weighting approach allows for a more accurate and relevant analysis of each company's financial performance. The weights or the market shares are calculated as follows:

(1) $$\mathrm{Market}\;\mathrm{shar}e_{\left(i\right)}=\frac{\mathrm{Sales}\;\mathrm{revenu}e_{\left(i\right)}}{\sum_{i\;\;=\;\;1}^{n}\;\mathrm{Sales}\;\mathrm{revenue}}\;\times 100$$

where n = the number of companies in each hypothetical market.

Net profit margin (NM) ratio is computed as follow:

(2) $$\mathrm{NM}\;(\text{\%})=\frac{\mathrm{Net}\;\mathrm{income}}{\mathrm{Total}\;\mathrm{sales}\;\mathrm{revenue}}\;\times 100$$

Weights are used to compute the weighted average net profit for each industry along the period from 2018 to 2021 as measured as follow:

(3) $$\mathrm{Weighted}\;\mathrm{average}\;\mathrm{NM}\;\mathrm{ratio}\;=\;(\overset{n}{\underset{i\;\;=\;\;1}{\sum }}\;{\mathrm{MS}}_{\left(i\right)}\;\times NM_{\left(i\right)})$$

N = number of the companies in the industry

MS = market share %

NM = net profit margin %

It should be mentioned that the financial information extracted from the WSJ comes in different currencies, depending on where the headquarter of the business is. The monetary units have been converted to Canadian dollars (CAD) for comparison purposes using historical annual exchange rates, which are extracted from Bank of Canada official website.^64^

Furthermore, two correlations have been studied using linear regression analysis. The number of employees and the market share are correlated to the net income. Linear regression analysis is frequently employed to determine relationships between variables.^65,66^ Equation (4) is used to express the regression line:

(4) Y=a+bX

where, Y = dependent variable, X = independent variable, a = intercept, and b = slope. The coefficient of determination (R^2^) of the derived equation displays how well the line fits the variables under investigation.^67,68^ An R^2^ value closer to 1.0 indicates a stronger linear relationship between the variables. The coefficient of determination (equation 5) was calculated using equation (5):

(5) $$R^{2}=\frac{{\mathrm{SS}}_{\mathrm{yy}}-\mathrm{SSE}}{{\mathrm{SS}}_{\mathrm{yy}}}$$

where SS_yy_ is the sum of the square deviations in the y variable, and SSE is the sum of the squared errors.^69^

## Result and discussion

### Weighted average net profit margin

**Figure 1.**
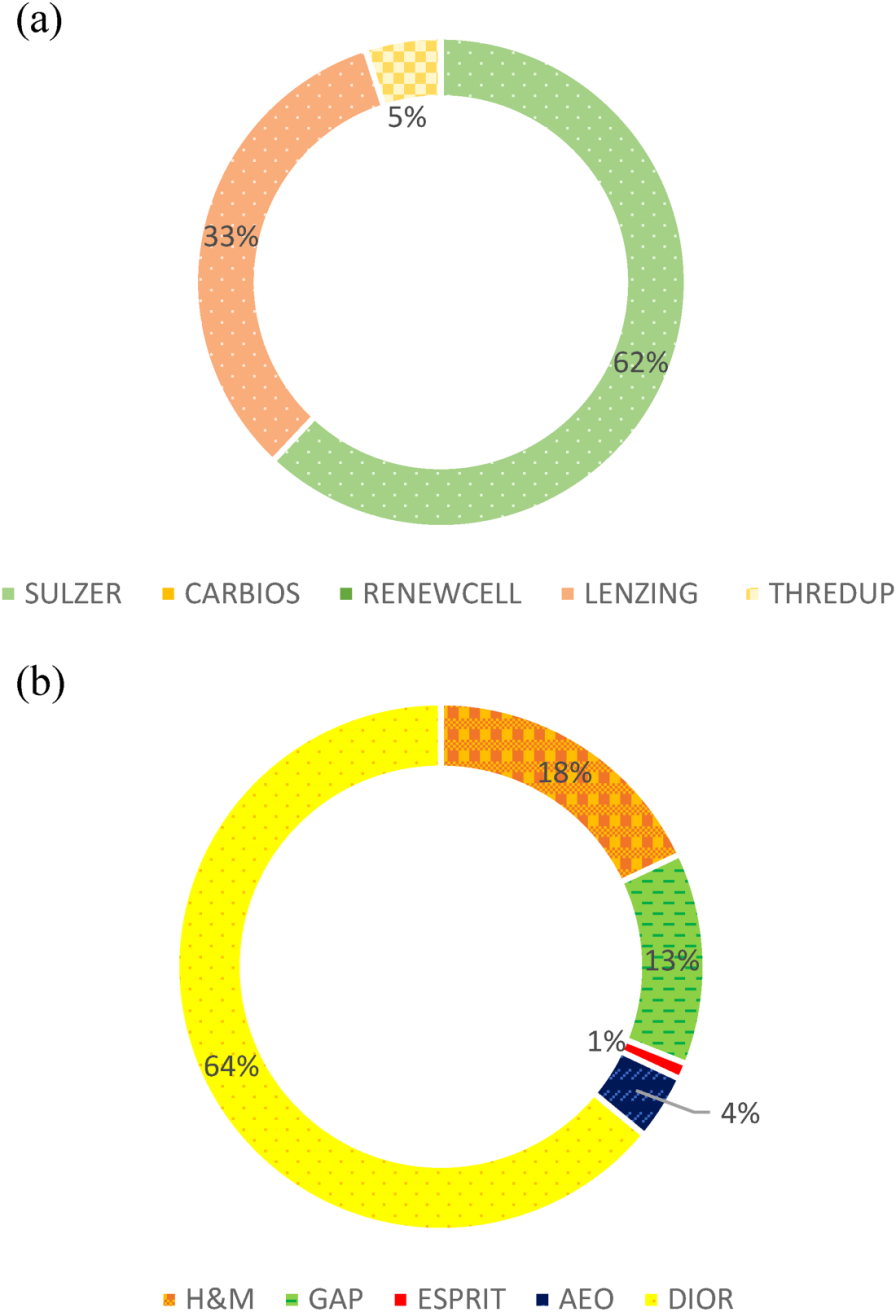
Market share of the selected companies in 2022: (a) sustainability fashion group and (b) conventional fashion group.

Figure 1 shows the market share for the selected sustainable and conventional fashion companies in 2022. The market leader for the sustainability fashion group is Sulzer (Figure 1(a)), with a market share of 62%. The next largest contributor is Lenzing, at 33%, about half of Sulzer. It appears that the sustainability fashion industry is emerging, and there are a few dominant players. The contributions from Carbios and Renewcell are negligible (<0.03%), and both are not visible in Figure 1(a).

CD is the leader in the conventional fashion group (Figure 1(b)), with a market share of 64% in 2022. The next largest contributor is H&M, at 18%, and approximately one-third the size of CD. Unlike the sustainability fashion group shown in Figure 1(a), the distribution of the market shares of the conventional fashion companies are more evenly distributed (Figure 1(b)). The finding suggests a relatively more established and mature industry sector.

It is worth noting that the total sales revenue of all the selected companies in the sustainability fashion group was about $7030 million CAD in 2022. On the contrary, the total sales revenue from the conventional group was nearly $162,200 million CAD in 2022. The conventional fashion companies studied in this study have a total sales revenue approximately 23 times higher than that of the sustainability brands. Given the rise of consumers’ sustainability movement,^37^ the result suggests that there is currently a sizeable potential market for the sustainability fashion companies.

**Figure 2.**
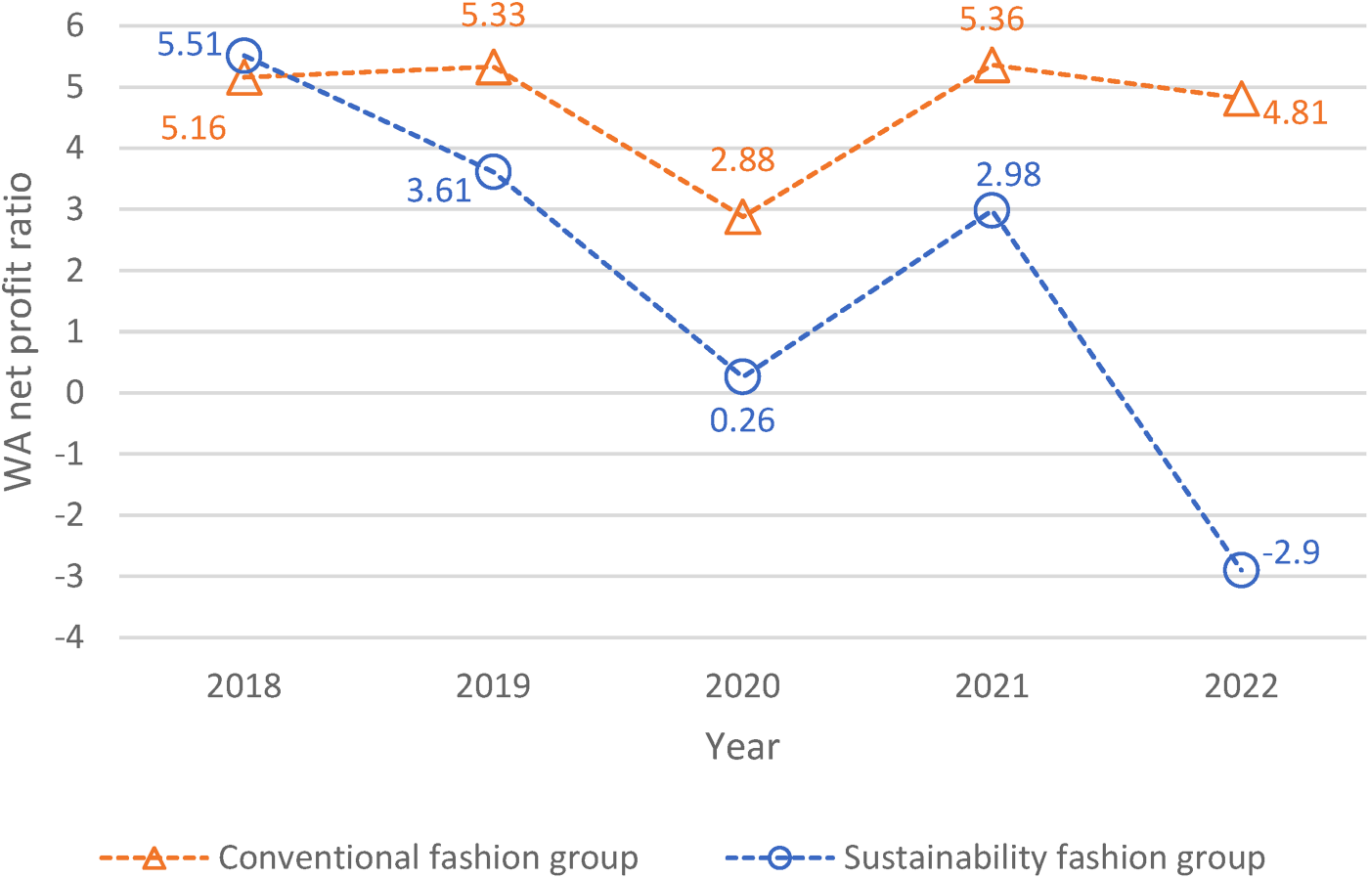
Evolution of weighted average (WA) net profit ratio from 2018 to 2022.

The market shares are used to calculate the weighted average (WA) net profit for each group. From Figure 2, starting from 2018 till 2022, the conventional fashion industry has a higher WA net profit ratio than the sustainability fashion industry. The WA net profit ratio of the conventional fashion industry averaging +4.7 during the 5-year study period. It is evident that the selected conventional fashion companies in this study are more profitable, on a weighted average basis, compared to the sustainability companies. This may be due, in part, to the much larger overall sales revenue of the fast fashion industry. However, the differences in business operations between the companies should be considered. For example, the conventional fashion group (i.e. CD, H&M, Esprit, AEO, and GAP) focuses mainly on designing, manufacturing, and selling clothing. On the other hand, the sustainability companies (i.e. Sulzer, Carbios, Renewcell, Lenzing, and Thredup) primarily focus on textile management and other byproducts derived from the manufacturing process. Specifically, Sulzer has partnered with H&M to control and develop “Worn Again,” a chemical recycling process for textiles.^70^ Given the nature of research and development projects, Sulzer's work may result in smaller profitability margins, especially considering the dynamic nature and development of chemical textile recycling. However, the implementation of these research and development projects is commendable, as they contribute directly the Sustainable Development Goal (SDG) 12 “Ensure sustainable consumption and production patterns.”

Over time, a decreasing trend is observed in the weighted average net profit margin ratio in the sustainability fashion group from +5.51 in 2018 to −2.90 in 2022. When COVID-19 hit, both industries’ profitability dropped from the previous year (see decrease from 2019 to 2020, Figure 2). In fact, the sustainability fashion group experienced about a 92% drop from between 2019 and 2020. On the other hand, the conventional fast fashion industry appeared more resilient, and dropped less than 45% in weighted average net profit ratio. The conventional fashion market was still in a better financial position during COVID-19 perhaps because they carried multiple lines of products and enjoyed higher flexibility in their business plans compared to their respective peers in the sustainability fashion group. The flexibility in operation allowed them to streamline their effort in a few specific apparel divisions such as leisurewear, which proved to be popular during and post-COVID. The difference in profit margin ratios between the groups was similar between 2019 and 2021 (Figure 2). In 2022, the conventional fast fashion industry profitability margin dropped by 10% compared to the previous year. On the other hand, the sustainability fashion companies faced the largest drop in net profit ratio during the entire study period. The sustainability fashion companies faced a negative profitability ratio (−2.90) in 2022, probably due in part to the increasing expenses near the end of the global pandemic. For instance, Lenzing, with around 33% market share among the sustainability fashion group (Figure 1(a)), faced a 64% growth in expenses between 2021 and 2022.^71^ This trend was also observed in the other companies within the sustainability group, perhaps due to the push for green recovery post-COVID. Galanakis et al.^72^ studied green recovery post COVID and concluded that bioeconomy could enhance its resilience and sustainability. Overall, the sustainability fashion group appears more sensitive to fluctuations in business expenses. The finding suggests more aid such as government initiatives and incentive programs may be beneficial to the growth of the sustainability fashion sector.

### Profitability gap between the groups

**Figure 3.**
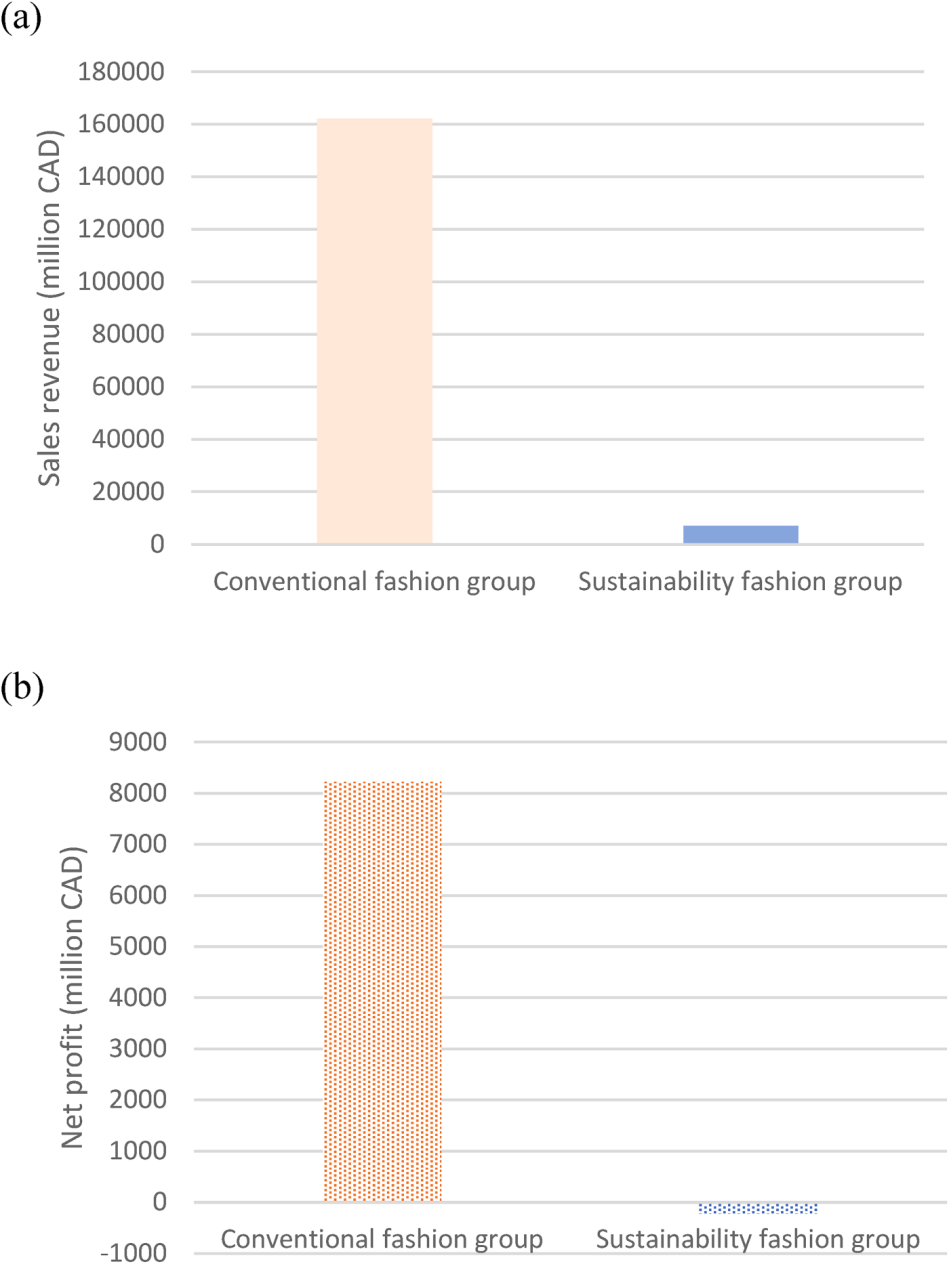
Comparisons of the (a) sales revenue and (b) net profit between the groups in 2022.

Figure 3 shows the profitability gap between the sustainability and the conventional fashion groups using the most recent available financial data. The conventional fashion companies have 23 times more sales revenue than the sustainability group in 2022 (Figure 3(a)). Net profit (Figure 3(b)) was negative for the sustainability group in 2022, indicating that subset of companies spent more money than they made. The findings suggest that the business characteristics of these fashion companies are very different. It is believed that the difference in sales revenue is the main contributing factor to this substantial gap. A low net profit can generally be explained by the following factors: low sales revenue, high operating expenses, high-interest expense, and strict governmental regulations.^73^ Government aid or other similar programs are recommended to grow the sustainability fashion companies. Results suggest that the five sustainability fashion companies considered in this study may be vulnerable to future public health emergencies and pandemic-related lockdowns. Currently, the roles of the government and regulatory bodies on SGD 12 are not clear, and more work is needed to improve sustainability of the sector. Specifically, the development of environmental sustainability awards, business tax relief programs, and educational campaigns are some possible options.

### Profitability in terms of entity size

**Figure 4.**
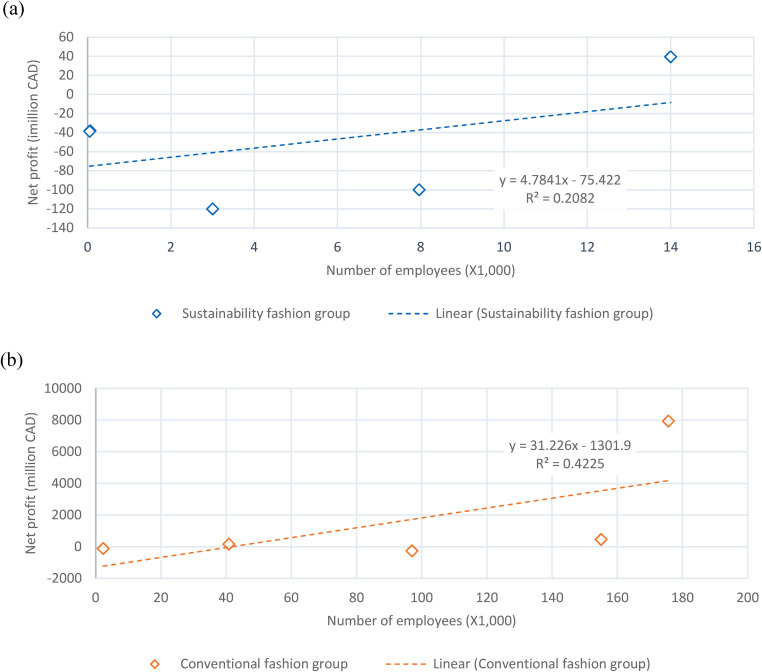
Correlation between company size and net profit in (a) sustainability fashion group and (b) conventional fashion group in 2022.

Figure 4(a) and (b) show the relationship of company size with net profit for the sustainability and the conventional fashion groups, respectively. Since all the selected companies are multinational corporations (Table 1) with offices located in different socio-economical regions, caution is needed to interpret the regression results. There is a weak but observable positive relationship (b =  + 4.8, R^2^ = 0.21) between net profit and the number of employees in the sustainability fashion group. A more obvious positive relationship (slope, b =  + 31.2, R^2^ = 0.42) is observed in the conventional fashion group. Results suggest that the larger company size may be beneficial to profitability in the fashion industry, at least based on the analysis of the 10 companies in this study. Sustainability fashion companies are recommended to recruit more employees. This is also consistent with the finding from Figure 2 that sustainability fashion companies studied are generally more dynamic and evolving. The R^2^ value is slightly higher in the conventional fashion group (Figure 4(b)), probably due to the larger workforce associated with a mature and well-established sector.

### Profitability in terms of market share

**Figure 5.**
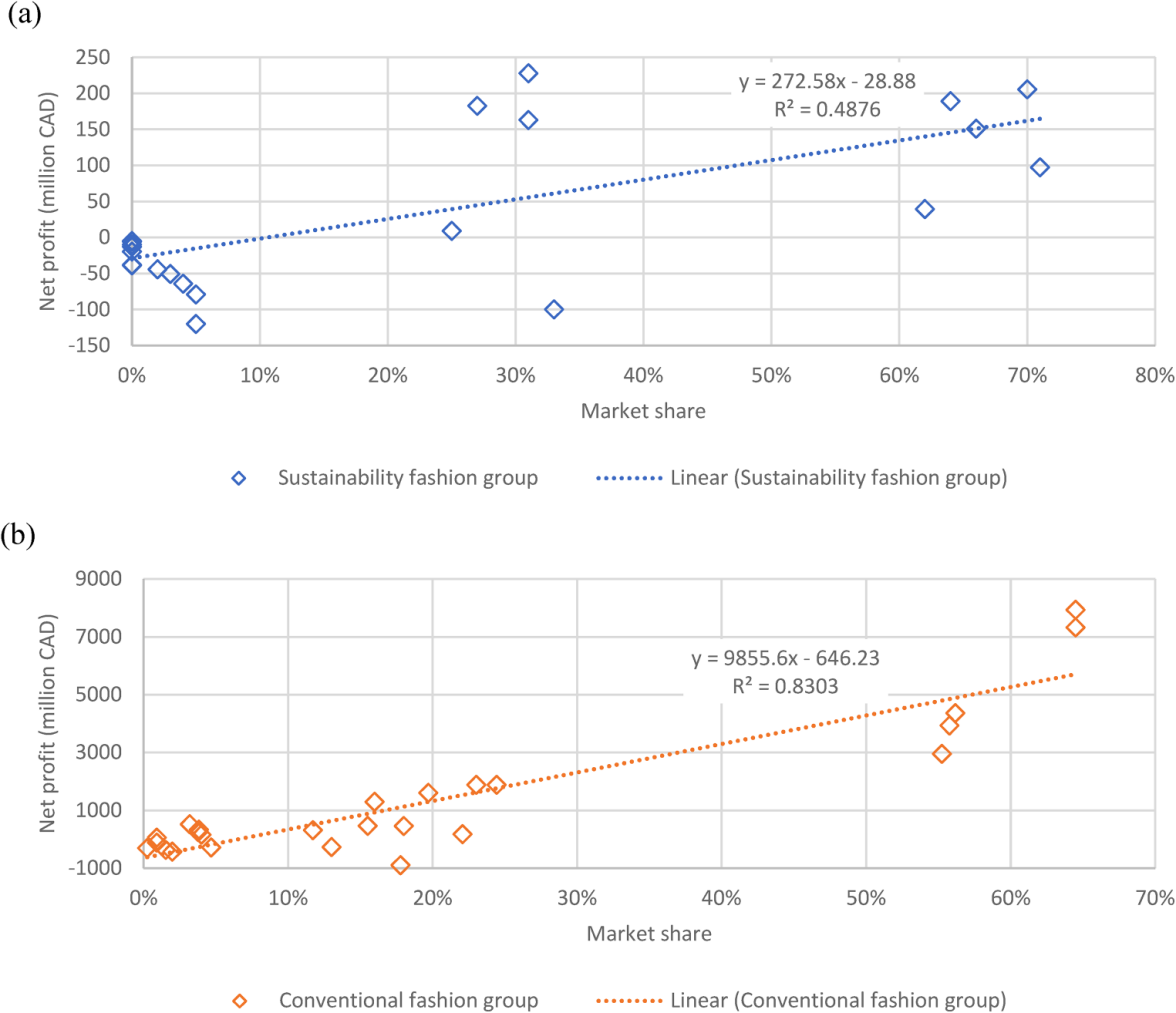
Correlation between businesses’ market shares and net profits for (a) sustainability fashion group and (b) conventional fashion group from 2018 to 2022.

In the context of the sustainability fashion group, the positive correlation observed between market share and net profit, as depicted in Figure 5(a), suggests that as companies increase their market share within this sector, their net profit also tends to rise. This alignment implies that consumers’ increasing preference for sustainable fashion products could be translated into improved financial performance for the companies operating in this segment. The moderate slope of +272.6 indicates that even incremental gains in market share may result in notable increases in net profit. However, a moderate R^2^ value of the correlation suggests that while there is a connection between market share and net profit, other factors beyond market share also contribute to financial outcomes within sustainability-oriented fashion businesses. Relatively higher data scattering is observed in Figure 5(a) than (b), suggesting an emerging and less established market for the sustainability fashion companies.

Conversely, the conventional fashion industry, as portrayed in Figure 5(b), showcases a substantially stronger positive correlation with a slope of +9855.6. The high R^2^ value of 0.83 indicates that an observable proportion of the variance in net profit can be attributed to changes in market share in this sector. This suggests that the competitive nature of the conventional fashion market, characterized by rapid shifts in consumer trends and preferences, has a significant impact on the financial performance of companies operating within it. The conventional fashion group's high sensitivity to market share fluctuations also highlights the potential risks of overreliance on short-term strategies, as market dynamics can rapidly change. It is recommended that organization led initiatives should be implement to promote sustainability practices in the conventional fashion industry. This is especially important for the few dominated companies in the sector, who enjoyed a noticeably higher net profit.

The differences between the two industries highlight that market share indeed affects profits, but in different ways. Conventional fashion companies require fast reactions to changing trends, where having a larger market share really matters. Conversely, companies in the sustainability fashion group might combine growing their market share with genuine sustainability and ethical branding.

### Limitations

A total of 10 companies are selected in this case study to examine the sustainability, profitability, and resiliency of the fashion industry during the pandemic. Only 5 years of temporal data are considered in this study. Given the short time period and the differences in the selected multinational corporations, caution is needed to interpret the numerical results.

## Conclusion

The fast fashion model, characterized by quick production cycles, low-cost garments, and high consumer demand, has led to significant environmental and social impacts. This study identifies the financial performance through weighted average net profit margin and business characteristics that affect the profitability between the sustainability fashion industry and the fast fashion industry. In 2022, unlike the sustainability fashion industry, the market shares of companies in the conventional fashion group were evenly distributed. The total sales revenue within the sustainability fashion group was 24 times less than that of the conventional fashion group, indicating room for improving profitability. The fast fashion industry displayed a higher weighted average net profit margin ratio compared to the sustainability fashion industry. This ratio remained relatively consistent from 2018 to 2022, ranging from +4.81 to +5.16. Conversely, the sustainability fashion industry observed a declining trend in its weighted average net profit margin ratio, dropping from 5.51 to −2.90 over the study period, particularly near the end of COVID-19 period in 2023. The sustainability fashion group appears to be less resilient, and may be more vulnerable to future public health emergencies.

The fast fashion industry got 40 times more in net profit and 23 times more in sales revenue than the sustainability fashion industry in 2022. This difference in net profit and sales revenue might have occurred between two fashion industries due to high cost of goods sold and other operating expenses and high-interest expense. Furthermore, both industry sustainability fashion industry and fast fashion industry have positive relation in between net profit and number of employees (slope, b =  + 4.8 and b =  + 31.2 accordingly) and net profit and market shares (slope, b =  + 272.6 and b =  + 9855.6). Notably, the correlation between net profit and company size in the sustainability and conventional fashion industry was less pronounced, respectively. In contrast, both the sustainability fashion and conventional fashion sectors showcased a strong association between net profit and market share, supported by R^2^ values of 0.49 and 0.83, respectively. This pattern underscores the pivotal role that market trends and consumer preferences play in shaping the financial trajectories of these industries. It appears that the fashion industry has more opportunities to be more profitable by adopting socially responsible goals.

## List of acronyms

CAD: Canadian dollars
ESG: Environmental, social, and governance
SDG: Sustainable development goal
SSE: Sum of the squared errors
WA: Weighted average
WSJ: Wall Street Journal
